# A Brain-Targeting Bispecific-Multivalent Antibody Clears Soluble Amyloid-Beta Aggregates in Alzheimer’s Disease Mice

**DOI:** 10.1007/s13311-022-01283-y

**Published:** 2022-08-08

**Authors:** Fadi Rofo, Silvio R. Meier, Nicole G. Metzendorf, Jamie I. Morrison, Alex Petrovic, Stina Syvänen, Dag Sehlin, Greta Hultqvist

**Affiliations:** 1 Department of Pharmacy, Uppsala University, 75124 Uppsala, Sweden; 2 Department of Public Health and Caring Sciences, Uppsala University, 75185 Uppsala, Sweden

**Keywords:** Multivalent antibodies, Bispecific, Aβ, Oligomers, BBB, Mouse models

## Abstract

Amyloid-β (Aβ) oligomers and protofibrils are suggested to be the most neurotoxic Aβ species in Alzheimer’s disease (AD). Hence, antibodies with strong and selective binding to these soluble Aβ aggregates are of therapeutic potential. We have recently introduced HexaRmAb158, a multivalent antibody with additional Aβ-binding sites in the form of single-chain fragment variables (scFv) on the N-terminal ends of Aβ protofibril selective antibody (RmAb158). Due to the additional binding sites and the short distance between them, HexaRmAb158 displayed a slow dissociation from protofibrils and strong binding to oligomers in vitro. In the current study, we aimed at investigating the therapeutic potential of this antibody format in vivo using mouse models of AD. To enhance BBB delivery, the transferrin receptor (TfR) binding moiety (scFv8D3) was added, forming the bispecific-multivalent antibody (HexaRmAb158-scFv8D3). The new antibody displayed a weaker TfR binding compared to the previously developed RmAb158-scFv8D3 and was less efficiently transcytosed in a cell-based BBB model. HexaRmAb158 detected soluble Aβ aggregates derived from brains of tg-ArcSwe and *App*^*NL−G−F*^ mice more efficiently compared to RmAb158. When intravenously injected, HexaRmAb158-scFv8D3 was actively transported over the BBB into the brain in vivo. Brain uptake was marginally lower than that of RmAb158-scFv8D3, but significantly higher than observed for conventional IgG antibodies. Both antibody formats displayed similar brain retention (72 h post injection) and equal capacity in clearing soluble Aβ aggregates in tg-ArcSwe mice. In conclusion, we demonstrate a bispecific-multivalent antibody format capable of passing the BBB and targeting a wide-range of sizes of soluble Aβ aggregates.

**Supplementary Information:**

The online version contains supplementary material available at 10.1007/s13311-022-01283-y.

## Introduction

Protein aggregation is one of the main pathological hallmarks in several neurodegenerative diseases. In Alzheimer’s disease (AD), the most common neurodegenerative disorder, the amyloid-beta peptide (Aβ) starts to aggregate and deposits extracellularly in the brain of the affected individuals. The aggregates of Aβ can be of different size and solubility, ranging from small soluble dimers and trimers to the intermediate soluble species (termed as oligomers and protofibrils) and to finally insoluble fibrils, which constitute the core components of the amyloid-plaques [1–3]. Different Aβ aggregates exhibit distinct toxicity mechanisms, with a growing body of evidence suggesting a correlation of oligomers and protofibrils with the neuronal and synaptic damage observed in AD [4–6]. Due to their small size and high mobility, soluble oligomers of Aβ are more likely to exert their neurotoxic effects through their ability to permeabilize and cross cell membranes [7, 8], while the slightly larger protofibrils have been shown to induce toxicity by other mechanisms, for example, by enhancing neuroinflammation [7]. In contrary to these findings, studies have demonstrated some neuroprotective effects of Aβ monomers [9, 10]. Thus, targeting of oligomers and protofibrils, without interaction with monomers, seems to be an advantageous therapeutic strategy. Furthermore, Aβ monomers exist at higher quantities in the periphery, where binding to the monomers can reduce the ability of the therapeutic molecule to reach intra-brain Aβ aggregates and could potentially also increase transport of peripheral monomers to the brain. Therefore, molecules that selectively and strongly bind Aβ oligomers and protofibrils have great diagnostic and therapeutic potential.

Antibody-based immunotherapy is one of the most promising therapeutic strategies for several diseases. In AD, a number of monoclonal antibodies targeting different species of Aβ have entered clinical trials [11]. Some of these antibodies have been discontinued due lack of efficacy and/or the associated adverse effects [12, 13]. Only one monoclonal anti-Aβ antibody (aducanumab) has been recently conditionally approved by the FDA [14], with three more antibodies (lecanemab, gantenerumab and donanemab) currently in phase 3 clinical trials [15–18]. To discriminate between binding to aggregated Aβ over monomers, these three antibodies (aducanumab, lecanemab and gantenerumab) utilize the avidity effect [19], meaning that both paratopes of the antibodies simultaneously bind to the targeted protein. Due to the spatial distance between the two paratopes, these antibodies bind strongly to aggregated protofibrils and/or fibrils, while displaying weak binding to monomers and small oligomers. Despite the flexibility of the two arms of antibodies, the distance between the paratopes of an IgG antibody is likely loo large to achieve high avidity binding to small oligomers [20–22]. Generation of antibodies that bind to all types of aggregates, and at the same time display weak binding only towards monomers, represents a big challenge for Aβ immunotherapy.

To improve antibody binding strength to the toxic aggregates of Aβ, we have recently designed and characterized a hexavalent antibody, HexaRmAb158 [23]. This multivalent antibody format has additional binding sites in the form of single chain fragment variable (scFv) recombinantly added to the N-terminal ends of RmAb158 antibody (an antibody with the same paratopes as lecanemab). Our previous work has shown that due to the additional binding sites, the dissociation time of HexaRmAb158 from Aβ protofibrils was significantly prolonged, resulting in a 40 times greater avidity compared to RmAb158. Further, the shorter distance between the additional binding sites enabled the hexavalent antibody to strongly bind smaller oligomers of Aβ, while retaining its high avidity binding to protofibrils and intermediate and weak binding preference to the insoluble fibrils and monomers, respectively. To date, the therapeutic significance of the hexavalent antibody format has been mainly investigated in vitro. However, to test the therapeutic application of this antibody format in vivo, further protein-engineering is required to make it possible for HexaRmAb158 to cross the blood–brain barrier (BBB) and to reach its intra-brain targets.

The BBB is another big challenge in the field of AD immunotherapy as the tightly connected endothelial cells of the BBB restrict the transport of macromolecules, such as antibodies, between the blood and brain. It has previously been demonstrated that less than 0.1% of the peripherally administered antibodies can pass the BBB [24, 25]. One way to shuttle antibodies over the BBB is via transferrin receptor (TfR)-mediated transcytosis. Antibodies binding to the TfR, such as the rat anti-mouse TfR antibody (8D3), have demonstrated the ability to pass the BBB in pre-clinical studies [26–28]. We have demonstrated an 80 times higher brain uptake of the RmAb158 antibody when two TfR binding moieties in the form of scFv (scFv8D3) were added to the C-terminal ends of the antibody’s light chains, forming the bispecific antibody RmAb158-scFv8D3 [29]. The BBB transporter (scFv8D3) has also been recombinantly linked to other full-length antibodies [30, 31], antibody fragments [32, 33] and peptides [34, 35], to improve their pharmacokinetic properties and/or diagnostic/therapeutic significance in pre-clinical mouse models of AD.

The aim of the current study was to develop a bispecific multivalent anti-Aβ oligomer antibody and to investigate its therapeutic potential in AD. Specifically, by recombinantly linking the BBB transporter (scFv8D3) to the hexavalent antibody format, we wanted to investigate the ability of the new antibody (HexaRmAb158-scFv8D3) to pass the BBB and to reduce brain levels of Aβ oligomers in transgenic mouse models of AD.

## Materials and Methods

### Antibody Design

**Fig. 1:**
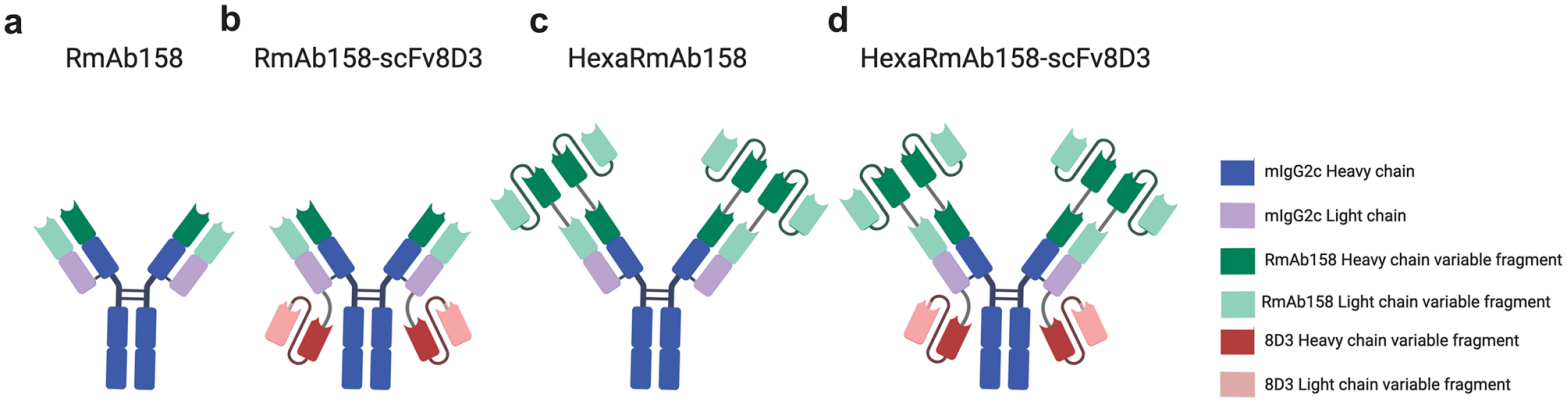
Design of the four recombinant antibodies. **a** RmAb158. **b** RmAb158-scFv8D3 where scFv8D3 is added to the C-terminal end of the RmAb158 light chains. **c** HexaRmAb158, where scFvs consisting of the heavy and light variable domains of RmAb158 are added to the N-terminal ends of RmAb158’s heavy and light chains. **d** HexaRmab158-scFv8D3, where scFv8D3 is added to the C-terminal ends of the HexaRmAb158 light chains

The antibodies were designed based on RmAb158 (Fig. 1a), a recombinant version of mAb158, i.e. an Aβ protofibril selective antibody and the murine parent of lecanemab [36]. The bispecific antibody RmAb158-scFv8D3 (Fig. 1b) was designed by recombinantly adding a scFv of the rat anti-mouse TfR antibody 8D3 to the C-terminal ends of RmAb158 light chains [29]. The scFv8D3 was added using the following linker (APGSGTGSAPG). HexaRmAb158 (Fig. 1c) was generated by recombinantly adding scFv consisting of the heavy and light variable domains of RmAb158 to the N-terminal ends of both the heavy and light chains of RmAb158, as described previously [23]. Using the above-mentioned linker (APGSGTGSAPG), scFv8D3 was added to the light chain C-termini of HexaRmAb158 to generate HexaRmAb158-scFv8D3 (Fig. 1d).

### Cloning, Expression and Purification

The heavy and light chains of the antibodies were cloned into separate pcDNA3.4 vectors (GeneArt, Regensburg, Germany). Human Expi293 cells were transiently transfected with 70% light chain plasmids and 30% heavy chain plasmids as described previously [23, 37]. Seven days after transfection, the cells were harvested, and the antibodies were purified using an Äkta start system with protein G columns (Cytiva, Uppsala, Sweden). After elution with acetic acid 0.7%, the buffer was exchanged to PBS using Zeba desalting columns (Thermo Scientific, Waltham, MA), and the antibodies were stored at −20 °C until further application.

### SDS-PAGE

Purified antibodies were mixed with LDS sample buffer (Thermo Scientific, Waltham, MA) without adding reducing agents. Samples were loaded onto 4–12% Bis–Tris protein gels (Thermo Scientific, Waltham, MA) and run for 2 h at 80 V using Bolt MES SDS running buffer (Thermo Scientific, Waltham, MA). The gel was washed three times with water, followed by staining with PAGE blue protein solution (Thermo Scientific, Waltham, MA) to analyse the size and purity of the eluted antibodies.

### Thermal Shift Assay

Structural stability of HexaRmAb158-scFv8D3 and RmAb158-scFv8D3 was evaluated as described previously [23]. The two antibodies (final concentration 1 μM) were heated in a glass capillary undergoing linear temperature increase from 35 to 90 °C using a Tycho nt.6 instrument (NanoTemper technologies, München, Germany), where fluorescence intensities were recorded at 330 nm and 350 nm.

### Indirect ELISA to Demonstrate HexaRmAb158-scFv8D3 Binding to TfR and Aβ

Ninety-six-well half-area plates (corning Inc., Corning, NY) were coated overnight at 4 °C with either 500 ng/mL of mouse TfR protein (recombinantly produced in the lab) or 10 nM of Aβ protofibrils, prepared as described previously [36]. The following day, the plates were incubated for 2 h at room temperature (RT) with ELISA blocking buffer (1% BSA in PBS), followed by incubation with serial dilutions of the purified antibodies for additional 2 h. A secondary anti-mouse IgG-HRP conjugated antibody (Sigma-Aldrich, Stockholm, Sweden) was used for detection, and signals were developed with K-blue aqueous TMB (Neogen Corp, Lexington, KY). The absorbance was measured at 450 nm using FLUOstar Omega microplate reader (BMG, Ortenberg, Germany). The plates were washed with 0.05% Tween-20 in PBS between each step.

### In Vitro BBB Transcytosis Assay

The previously described in vitro BBB transcytosis assay was used [38]. In this assay, the murine cerebral endothelial cell line (cEND from ABM T0290) (passages 12–23) was grown on rat tail Collagen Type I (Sigma, 50 μg/ml) coated 75 cm^2^ culture flasks (Sarstedt, Numbrecht, Germany) in complete cEND medium (DMEM supplemented with 10% FBS, 1X non-essential amino acids, 1X Glutamax, 1 mM sodium pyruvate and 10 U/ml penicillin/streptomycin—all media and supplements were purchased from Gibco) at 37 °C and 5% CO_2_. For all transcytosis assays, ThinCert™ translucent PET membranes with high density 0.4 um pores (1 × 10^8^ pores/cm^2^) were used in 24-well cell culture plates (Greiner, Kremsmünster, Austria). Apical chambers of the Greiner hanging inserts were coated with Collagen type IV (Fisher Scientific, 20 μg/cm^2^) followed by fibronectin (Sigma, 20 μg/cm^2^), each incubation lasting for one hour at 37 °C and 5% CO_2_. The cEND cells were plated at a density of 9 × 10^4^ cells per transwell in a final volume of 125 μl in the apical chamber on day one. Four hours later, the apical and basolateral medium was changed to differentiation medium (2% FBS, 1X non-essential amino acids, 1 × Glutamax, 1 mM sodium pyruvate and 10 U/ml penicillin/streptomycin). On day four, the Greiner membranes were pulse-incubated apically with either 1 pmol or 20 pmol of RmAb158-scFv8D3 and HexaRmAb158-scFv8D3 antibodies in serum-free medium (no FBS) at 37 °C and 5% CO_2_ for 1 h. Volumes used for the pulse apical (75 µl) and basolateral chambers (400 µl) were collected to corroborate the starting concentration of the antibodies used and the voracity of the endothelial cell barrier, respectively. The monolayers were washed at RT in serum-free medium apically (400 μl) and basolaterally (800 μl) three times, with the final wash collected to monitor efficiency of removal of the unbound antibodies. Serum-free medium was added to the apical (75 μl) and basolateral (400 μl) chambers, and the cultures were incubated at 37 °C and 5% CO_2_ for 4 or 12 h. At these times, samples from apical and basolateral volumes were collected to assess the recycling and transcytosis of the antibodies into the apical and basolateral chambers, respectively.

### Sandwich ELISA Analysis of Cell Medium Samples from the Transcytosis Assay

Ninety-six-well half-area plates (corning Inc., Corning, NY) were coated with goat-anti mouse IgG, F(ab’)_2_ fragment specific antibody (Jackson ImmunoResearch, Cambridge, UK) diluted in PBS and incubated at 4 °C overnight. The wells were blocked for 1 h at RT with ELISA blocking buffer (1% BSA in PBS). Diluted and undiluted apical and basolateral samples from the transcytosis assay, along with known standard concentrations of RmAb158-scFv8D3 and HexaRmAb158-scFv8D3, were added to the wells and incubated for 2 h at RT. The wells were then incubated with a secondary goat anti-mouse IgG-HRP conjugated antibody (Sigma-Aldrich, Stockholm, Sweden). The signals were developed as described in the previous ELISA section.

### Labelling of Antibodies with 125-Iodine

Labelling of the antibodies with 125-iodine (^125^I) was performed as previously described [23, 39]. Equimolar amounts of HexaRmAb158-scFv8D3 and RmAb158-scFv8D3 were labelled with 16 MBq of ^125^I (Perkin Elmer Inc., Waltham, MA), resulting in a labelling yield of 75% and a specific activity of 70 MBq/nmol. For the therapeutic study, the radiolabelled antibody was mixed with the unlabelled antibody at a 1:9 ratio.

### Animals

The AD transgenic mouse model (tg-ArcSwe), harbouring the Swedish (KM670/671NL) and Arctic (E693G) APP mutations [40], and the AD knock-in mouse model (*App*^*NL−G−F*^) harbouring the Swedish, Arctic and Iberian APP mutations [41] were used in this study. Both lines were kept on a C57bl6 background. Littermates served as wild-type (WT) controls, and both females and males were included. All animal experiments were carried out following the ethical guidelines and having the ethical permission numbers: 5.8.18–13,350/17 and 5.8.18–20,401/20.

### Biodistribution Study in WT Mice

C57Bl/6 WT mice (6 months old) were intravenously injected via the tail vein with a tracer dose of 0.05 mg/kg of [^125^I]HexaRmAb158-scFv8D3 (*n* = 5) or [^125^I]RmAb158-scFv8D3 (*n* = 5). Two hour post injection, mice were euthanized by transcardial perfusion with 0.9% NaCl. Brains were dissected and immediately snap frozen on dry ice. Terminal blood was collected, with plasma separated from the blood cells (referred to as blood pellet throughout the study) by centrifugation at 10,000 × g for 10 min. Radioactivity in brain, blood samples and peripheral organs was measured using Wizard 2470 gamma counter (Perkin Elmer Inc., Waltham, MA) as described previously [34].

### Therapeutic Study in AD Mice

Tg-ArcSwe mice (10–11 months old) were intravenously injected via the tail vein with a therapeutic dose of 5 mg/kg (24 nmol/kg) of [^125^I]RmAb158-scFv8D3 (*n* = 5) or its equimolar equivalent of 7.5 mg/kg of [^125^I]HexaRmAb158-scFv8D3 (*n* = 5). The control group was injected with PBS (*n* = 4). Blood samples were obtained after 1 h, 4 h, 6 h, 24 h, 48 h and 72 h. At the terminal time point (72 h post injection), mice were euthanized, and brains, blood and peripheral organs were collected as described above. Radioactivity in brain, blood and peripheral organs was measured as described in the previous section. Brains were homogenized at 1:5 weight/volume ratio with Tris-buffered saline (TBS) and centrifuged for 1 h at 16, 000 × g at 4 °C to generate soluble brain extracts. The remaining pellet was homogenized at 1:5 weight/volume ratio with TBS containing 1% Triton-X (TBS-T) and centrifuged for 1 h at 16, 000 × g at 4 °C to generate soluble membrane-bound brain extracts. Finally, the remaining pellet was homogenized with 1:5 weight/volume ratio with 70% formic acid (FA) and centrifuged for 1 h at 16, 000 × g at 4 °C to generate the insoluble brain extracts. All the homogenization buffers contained cOmplete™ protease inhibitors (Roche, Basel, Switzerland). Aliquots (150–200 μL) from TBS and TBS-T brains extracts were ultra-centrifuged at 100,000 × g and 200,000 × g for 1 h at 4 °C to generate soluble extracts containing smaller protein aggregates. The generated extracts were stored at −80 °C until further application. A similar study was also performed in *App*^*NL−G−F*^ mice (5.5 months old) with the exception that a therapeutic dose of 2.5 mg/kg (12 nmol/kg) of [^125^I]RmAb158-scFv8D3 (*n* = 8) or its equimolar equivalent of 3.75 mg/kg of [^125^I]HexaRmAb158-scFv8D3 (*n* = 9) was given.

### Sandwich Aβ ELISA

Concentration of soluble Aβ aggregates was measured in the generated brain extracts as described previously [42]. Ninety-six-well half-area plates (corning Inc., Corning, NY) were coated overnight at 4 °C with 1 μg/mL 3D6 antibody (recombinantly produced in the lab). The plates were blocked with 1% BSA in PBS for 3 h at RT. TBS and TBS-T soluble brain extracts were diluted in ELISA incubation buffer (0.1% BSA, 0.05% Tween in PBS) and added to the plates. The plates were incubated overnight at 4 °C. Biotinylated 3D6 antibody (recombinantly produced in the lab) and streptavidin-HRP (Mabtech, Stockholm, Sweden) were used for the detection. Signals were developed as described in the previous sections. To assess and compare the binding of HexaRmAb158 and RmAb158 to biologically generated Aβ aggregates, the same ELISA setup was applied, with the exception that HexaRmAb158 or RmAb158 were used as the capture antibodies instead of 3D6.

### MSD Assays

Total Aβ concentration (Aβ38, Aβ40 and Aβ42) in the FA brain extracts was measured using the V-PLEX Aβ peptide panel 1 (6E10) kit, according to the manufacturer protocol (K15200G, Meso Scale Diagnostics, Rockville, MD). Concentrations of cytokines in the TBS soluble brain extracts and plasma samples were quantified using V-PLEX proinflammatory panel 1 mouse kit, according to the manufacturer protocol (K15048D, Meso Scale Diagnostics, Rockville, MD). For Aβ quantification, extracts were diluted at 1:3000 volume/volume ratio. While for cytokine quantification, TBS soluble extracts and plasma samples were diluted at 1:2 volume/volume ratio.

### Statistical Analysis

Data were analysed with either unpaired *t* test or one-way ANOVA followed by Bonferroni’s post-hoc analysis. Results are reported as mean ± SD. (*: *p* < 0.05, **: *p* < 0.01, ***: *p* < 0.001, n.s: *p* > 0.05).

## Results

### In Vitro Characterization of HexaRmAb158-scFv8D3

**Fig. 2:**
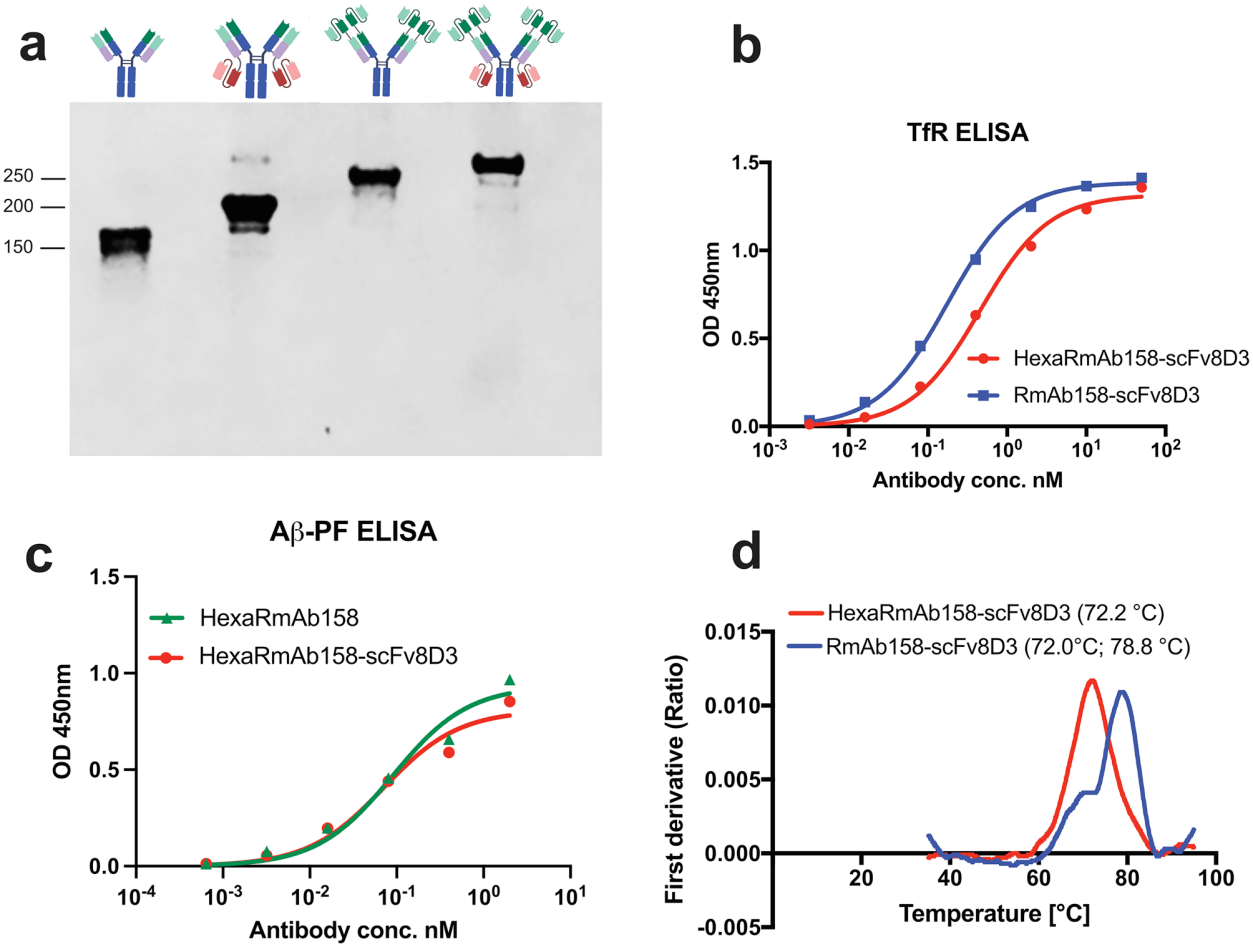
In vitro characterization of HexaRmAb158-scFv8D3. **a** SDS-PAGE showing a band at around 150 kDa for RmAb158, 200 kDa for RmAb158-scFv8D3, 250 kDa for Hexa-RmAb158 and 300 kDa for HexaRmAb158-scFv8D3. The complete SDS-PAGE can be found in Supplementary Fig. 5. **b** Indirect ELISA displaying binding of HexaRmAb158-scFv8D3 to mouse TfR. Binding to mouse TfR was twofold weaker with HexaRmAb158-scFv8D3 (EC50 = 6.3 nM) compared to RmAb158-scFv8D3 (EC50 = 3.2 nM). **c** Indirect ELISA displaying binding of HexaRmAb158-scFv8D3 to Aβ protofibrils. Similar binding to Aβ protofibrils was observed for HexaRmAb158 and HexaRmAb158-scFv8D3, demonstrating retained functionality despite the additional binding sites of HexaRmAb158-scFv8D3. **d** Structural stability of HexaRmAb158-scFv8D3 and RmAb158-scFv8D3 under thermal stress measured with Tycho nt.6 instrument

Following the recombinant production of the four antibodies, SDS-PAGE analysis was used to estimate their size and purity. A single band at approximately 150 kDa for RmAb158, 200 kDa for RmAb158-scFv8D3, 250 kDa for HexaRmAb158 and 300 kDa for HexaRmAb158-scFv8D3 was detected (Fig. 2a). Binding of HexaRmAb158-scFv8D3 to TfR and Aβ aggregates was assessed using an indirect ELISA setup. HexaRmAb158-scFv8D3 displayed functional binding to both TfR and Aβ protofibrils (PF), respectively (Fig. 2b, c). Noteworthy, HexaRmAb158-scFv8D3 displayed a weaker binding to TfR with an EC50 of 6.3 nM (Fig. 2b), which was 2 times higher than for RmAb158-scFv8D3 (EC50 3.2 nM). Regarding its structural stability under thermal stress, HexaRmAb158-scFv8D3 yielded an inflection temperature of 72.2 °C (Fig. 2d), similar to what has been previously reported for HexaRmAb158 [23]. RmAb158-scFv8D3 yielded two inflection temperatures (72 °C and 78.8 °C) (Fig. 2d). The inflection temperatures detected at 72 °C are likely due to the presence of scFv, which are more prone to thermal stress compared to full IgG antibodies [23].

### In Vitro BBB Transcytosis of HexaRmAb158-scFv8D3

**Fig. 3:**
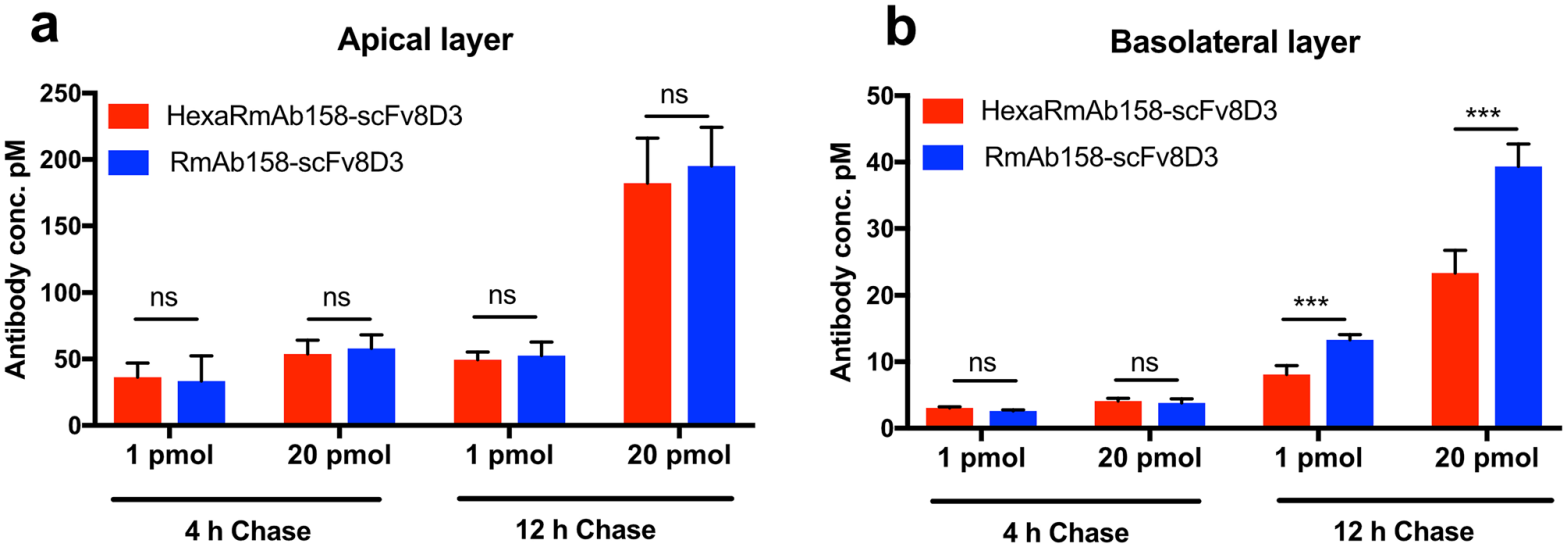
Graphical representations comparing the concentration of RmAb158-scFv8D3 and HexaRmAb158-scFv8D3 in the apical (**a**) and basolateral (**b**) compartments of the in vitro BBB transcytosis assay. Statistically significant decreases in the transport across the cEND cells into the basolateral layer were detected for HexaRmAb158-scFv8D3 compared to RmAb158-scFv8D3, both at low (1 pmol) and high (20 pmol) doses, only after 12 h chase. Results are presented as mean ± SD and analysed with unpaired t-test. (*: *p* < 0.05, **: *p* < 0.01, ***: *p* < 0.001, n.s: *p* > 0.05) (*n* = 6 per treatment)

BBB transcytosis of HexaRmAb158-scFv8D3 was investigated in vitro using a confluent monolayer of differentiated cEND cells in a transwell system, using RmAb158-scFv8D3 as a control. No significant differences in recycling into the apical compartment were detected between HexaRmAb158-scFv8D3 and RmAb158-scFv8D3 (Fig. 3a). However, results revealed a significant decrease in the transport across the cEND cells into the basolateral layer using HexaRmAb158-scFv8D3 compared to RmAb158-scFv8D3, selectively after 12 h chase, both at low (1 pmol) and high (20 pmol) doses (Fig. 3b). Despite this, HexaRmAb158-scFv8D3 showed a five- to tenfold higher transcytosis level compared to unmodified IgG antibodies without the scFv8D3 moiety at 12 h chase.

### ELISA Detection of Soluble Aβ Aggregates in Brain Homogenates Using HexaRmAb158

**Fig. 4:**
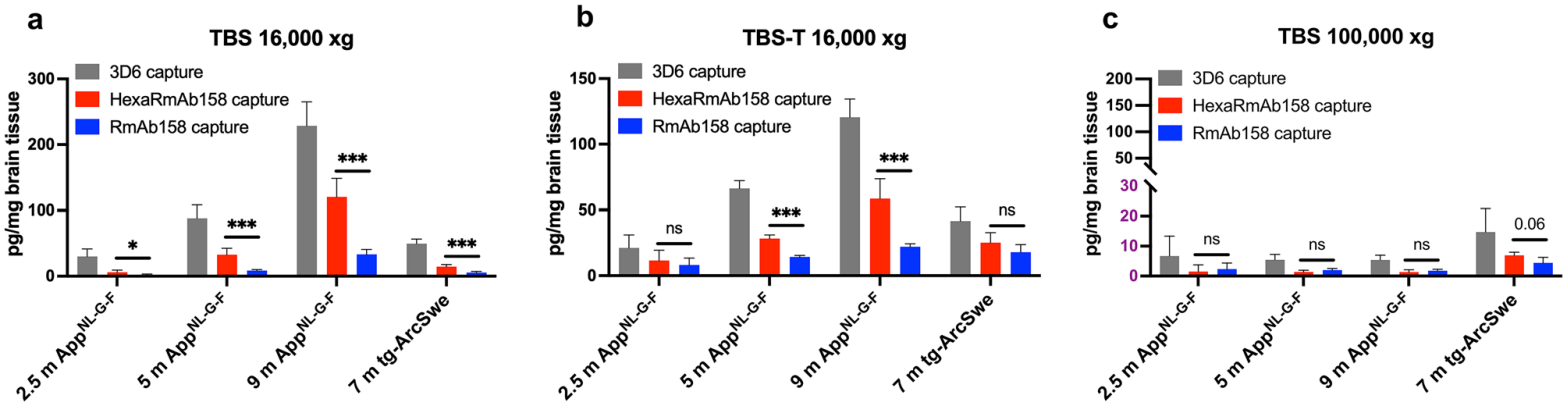
HexaRmAb158 and RmAb158 sandwich ELISA for the measurement of soluble Aβ aggregates in brain homogenates from *App*^*NL−G−F*^ mice (2.5, 5 and 9 months old) and tg-ArcSwe mice (7 months old), using 3D6 ELISA as a control. **a** HexaRmAb158 ELISA detected significantly higher concentrations of soluble Aβ aggregates compared to RmAb158 ELISA in TBS treated brain homogenates. **b** HexaRmAb158 ELISA detected significantly higher concentrations of Aβ compared to RmAb158 ELISA in TBS-Triton (TBS-T) brain homogenates from 5-month-old and 9-month-old *App*^*NL−G−F*^ mice. **c** No significant differences in Aβ concentrations were detected by HexaRmAb158 and RmAb158 ELISAs in TBS-brain homogenates ultra-centrifuged at 100 000 × g. HexaRmAb158 displayed a trend (*p* = 0.06) towards increased detection of Aβ aggregates in tg-ArcSwe brain TBS homogenates ultra-centrifuged at 100 000 × g. Results are presented as mean ± SD. To detect statistically significant differences between HexaRmAb158 and RmAb158 ELISAs, unpaired *t* test was applied. (*: *p* < 0.05, **: p < 0.01, ***: *p* < 0.001, n.s: *p* > 0.05) (*n* = 6 for 2.5 m *App*^*NL−G−F*^, *n* = 10 for 5 m *App*^*NL−G−F*^, *n* = 7 for 9 m *App*.^*NL−G−F*^, *n* = 6 for 7 m tg-ArcSwe)

Binding of HexaRmAb158 to in vivo generated soluble Aβ aggregates was evaluated by measuring the concentration of soluble Aβ aggregates in brain homogenates from Aβ overexpressing mice. A sandwich ELISA setup was established, where HexaRmAb158 [23], RmAb158 [36] or 3D6 [43] were used as the capture antibodies. The 3D6 antibody binds without avidity and equally strong to all Aβ species including monomers. Concentration of soluble Aβ aggregates was measured in brain homogenates prepared from *App*^*NL−G−F*^ mice of three different ages (2.5, 5 and 9 months old) and tg-ArcSwe mice (7 months old). Compared to RmAb158, HexaRmAb158 captured significantly more soluble Aβ aggregates in all brain tissues homogenized with TBS and centrifuged at 16 000 × g (Fig. 4a). The use of HexaRmAb158 as the coating antibody also detected a higher Aβ concentration in brain tissues homogenized with TBS-Triton and centrifuged at 16 000 × g, in 5 and 9 months old *App*^*NL−G−F*^ mice (Fig. 4b). No statistically significant differences in levels of soluble Aβ aggregates were identified when HexaRmAb158 and RmAb158 coated plates were used in the analysis of *App*^*NL−G−F*^ TBS brain extracts ultra-centrifuged at 100 000 × g (Fig. 4c), which contain a higher proportion of small Aβ aggregates than the samples centrifuged at 16 000 × g. Noteworthy, HexaRmAb158 showed a trend (*p* = 0.06) towards increased Aβ detection in tg-ArcSwe TBS brain extract centrifuged at 100 000 × g (Fig. 4c). In all these ELISAs, 3D6 captured more soluble Aβ aggregates in the different brain extracts compared to HexaRmAb158 and RmAb158 (Fig. 4).

### In Vivo Brain Uptake of HexaRmAb158-scFv8D3

**Fig. 5:**
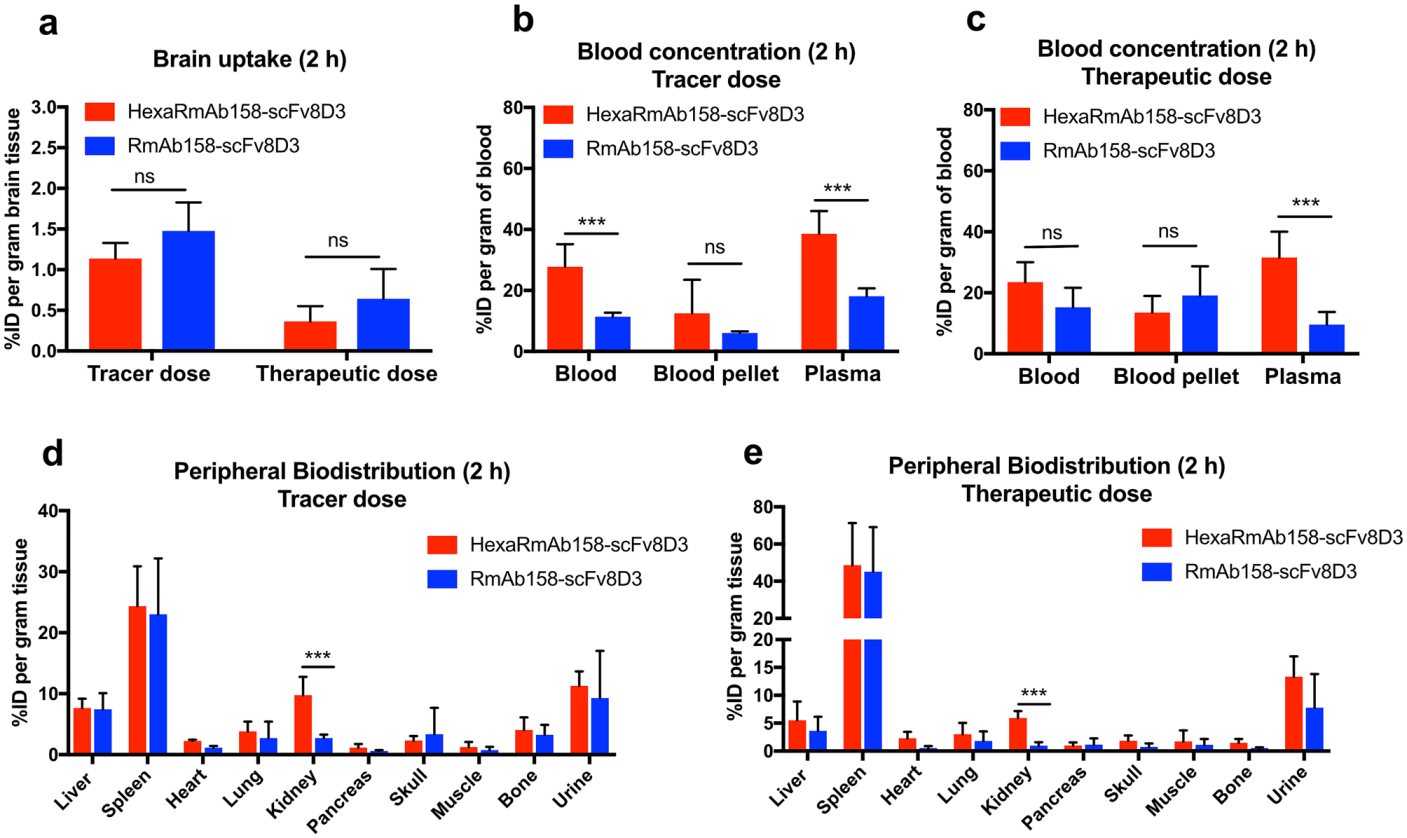
Comparison of [^125^I]HexaRmAb158-scFv8D3 and [^125^I]RmAb158-scFv8D3 concentrations in the brain and blood of 6-month-old C57Bl/6 WT mice. **a** Brain concentrations of [^125^I]HexaRmAb158-scFv8D3 and [^125^I]RmAb158-scFv8D3, expressed as percentage of injected dose (% ID) per gram brain tissue, 2 h post injection. **b** Statistically significant increase in blood and plasma concentrations of [^125^I]HexaRmAb158-scFv8D3 compared to [^125^I]RmAb158-scFv8D3, 2 h post injection, when given as a tracer dose. **c** Statistically significant increase in plasma concentrations of [^125^I]HexaRmAb158-scFv8D3 compared to [^125^I]RmAb158-scFv8D3, 2 h post injection, when given as a therapeutic dose. **d** and **e** Significantly higher concentrations of [^125^I]HexaRmAb158-scFv8D3 in the kidneys compared to [^125^I]RmAb158-scFv8D3, 2 h post injection, given at tracer and therapeutic doses respectively. Results are presented as mean ± SD and analysed with unpaired *t* test. (*: *p* < 0.05, **: *p* < 0.01, ***: *p* < 0.001, n.s.: *p* > 0.05) (*n* = 5 per group)

Brain delivery of HexaRmAb158-scFv8D3 was studied in WT mice (6 months old) using RmAb158-scFv8D3 as a positive control. Iodine-125 labelled antibodies, [^125^I]HexaRmAb158-scFv8D3 and [^125^I]RmAb158-scFv8D3, were intravenously administered at tracer dose (0.05 mg/kg; corresponding to 0.24 nmol/kg) or therapeutic dose (12 nmol/kg). Brain concentrations at 2 h, expressed as percentage of the injected dose (% ID) per gram brain tissue, were 1.13% and 0.36% when [^125^I]HexaRmAb158-scFv8D3 was administered at tracer and therapeutic doses, respectively (Fig. 5a). For [^125^I]RmAb158-scFv8D3, brain concentrations were 1.47% and 0.64% when administered at tracer and therapeutic doses, respectively (Fig. 5a). The difference in brain concentrations between [^125^I]HexaRmAb158-scFv8D3 and [^125^I]RmAb158-scFv8D3 was not statistically significant. However, a trend (*p* = 0.12) towards a lower brain uptake was observed for [^125^I]HexaRmAb158-scFv8D3 compared to [^125^I]RmAb158-scFv8D3 at tracer dosing. Interestingly, terminal plasma concentrations at 2 h were significantly higher in mice injected with [^125^I]HexaRmAb158-scFv8D3 compared to the plasma concentrations measured in mice administered with [^125^I]RmAb158-scFv8D3, both at tracer dose (Fig. 5b) and therapeutic dose (Fig. 5c). The two antibodies demonstrated a similar biodistribution to peripheral organs in WT mice at 2 h post injection, both at tracer dose (Fig. 5d) and therapeutic dose (Fig. 5e). The only detectable differences were seen in the kidneys, with significantly higher concentrations which were observed for [^125^I]HexaRmAb158-scFv8D3 compared to RmAb158-scFv8D3 (Fig. 5d, e).

### Brain Retention, Blood Half-Life and Peripheral Biodistribution of HexaRmAb158-scFv8D3 in AD Mice

**Fig. 6:**
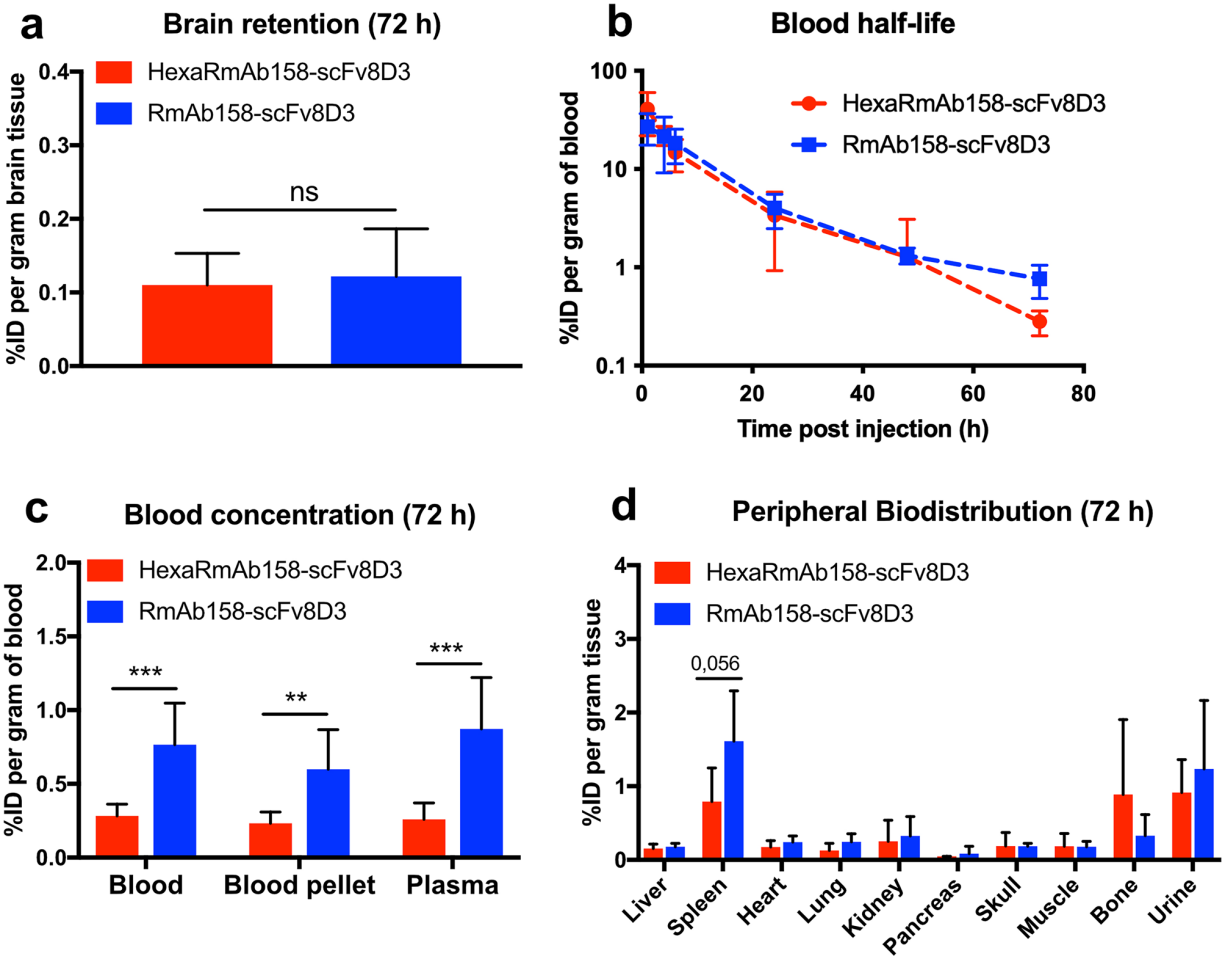
Biodistribution of [^125^I]HexaRmAb158-scFv8D3 and [^125^I]RmAb158-scFv8D3 in 10–11 months old tg-ArcSwe mice at 72 h post injection of a therapeutic dose of 24 nmol/kg. **a** Brain retention of [^125^I]HexaRmAb158-scFv8D3 and [^125^I]RmAb158-scFv8D3 in tg-ArcSwe mice, expressed as percentage of injected dose (% ID) per gram brain tissue, 72 h post injection. No statistically significant differences were detected between the two groups. **b** Blood concentrations of the two antibodies measured 1–72 h post injection. **c** Blood concentrations of [^125^I]HexaRmAb158-scFv8D3 were lower compared those of [^125^I]RmAb158-scFv8D3 at 72 h post injection. **d** Similar retention of [^125^I]HexaRmAb158-scFv8D3 and [^125^I]RmAb158-scFv8D3 in the peripheral organs, 72 h post injection. A trend towards a lower retention in the spleen was detected for [^125^I]HexaRmAb158-scFv8D3. Results are presented as mean ± SD and analysed with unpaired t-test. (*: *p* < 0.05, **: *p* < 0.01, ***: *p* < 0.001, n.s.: *p* > 0.05) (*n* = 5 per group)

Therapeutic doses of 7.5 mg/kg of [^125^I]HexaRmAb158-scFv8D3 (corresponding to around 24 nmol/kg, Mw 316 kDa) and 5 mg/kg of [^125^I]RmAb158-scFv8D3 (corresponding to around 24 nmol/kg, Mw 202 kDa) were administered to 10–11-month-old tg-ArcSwe mice. Retention of the antibodies in the brain, blood and peripheral organs was studied at 72 h post injection. Similar brain retention was observed between [^125^I]HexaRmAb158-scFv8D3 and [^125^I]RmAb158-scFv8D3 in these mice (Fig. 6a). Antibody retention was also evaluated following the generation of the different brain extracts of soluble (TBS), membrane-bound (TBS-T) and insoluble (FA) Aβ. Both antibodies were mainly present in the FA-soluble extracts, suggesting their retention mainly within the plaques area (Supplementary Fig. 1). Interestingly, the blood half-life of [^125^I]HexaRmAb158-scFv8D3 based on the elimination phase (from 24 to 72 h) was 12.5 h (Fig. 6b). This is a shorter blood half-life compared to that of [^125^I]RmAb158-scFv8D3, which was estimated to be around 21 h, i.e. similar to what has been previously reported [29, 44]. Interestingly, despite the higher blood concentration detected for [^125^I]HexaRmAb158-scFv8D3 at the early time points (Fig. 6b), the blood concentration of this antibody was significantly lower at the 72 h time point compared to the concentration of [^125^I]RmAb158-scFv8D3 (Fig. 6c). The two antibodies demonstrated a similar biodistribution in the peripheral organs of the tg-ArcSwe mice at 72 h post injection (Fig. 6d). Noteworthy, a trend to a lower concentration in spleen (*p* 0.056) was detected for [^125^I]HexaRmAb158-scFv8D3 compared to [^125^I]RmAb158-scFv8D3 (Fig. 6d). Biodistribution of [^125^I]HexaRmAb158-scFv8D3 was also studied in *App*^*NL−G−F*^ and found to be similar to that of tg-ArcSwe mice (Supplementary Fig. 2). [^125^I]HexaRmAb158-scFv8D3 demonstrated similar brain and peripheral organ retention to [^125^I]RmAb158-scFv8D3 (Supplementary Fig. 2a–c). Similar to results obtained in tg-ArcSwe mice, the concentration of [^125^I]HexaRmAb158-scFv8D3 in *App*^*NL−G−F*^ plasma was significantly lower than that of [^125^I]RmAb158-scFv8D3 at 72 h post injection (Supplementary Fig. 2b).

### Therapeutic Significance of HexaRmAb158-scFv8D3 in Lowering Soluble Aβ Aggregate Concentration in AD Mice

**Fig. 7:**
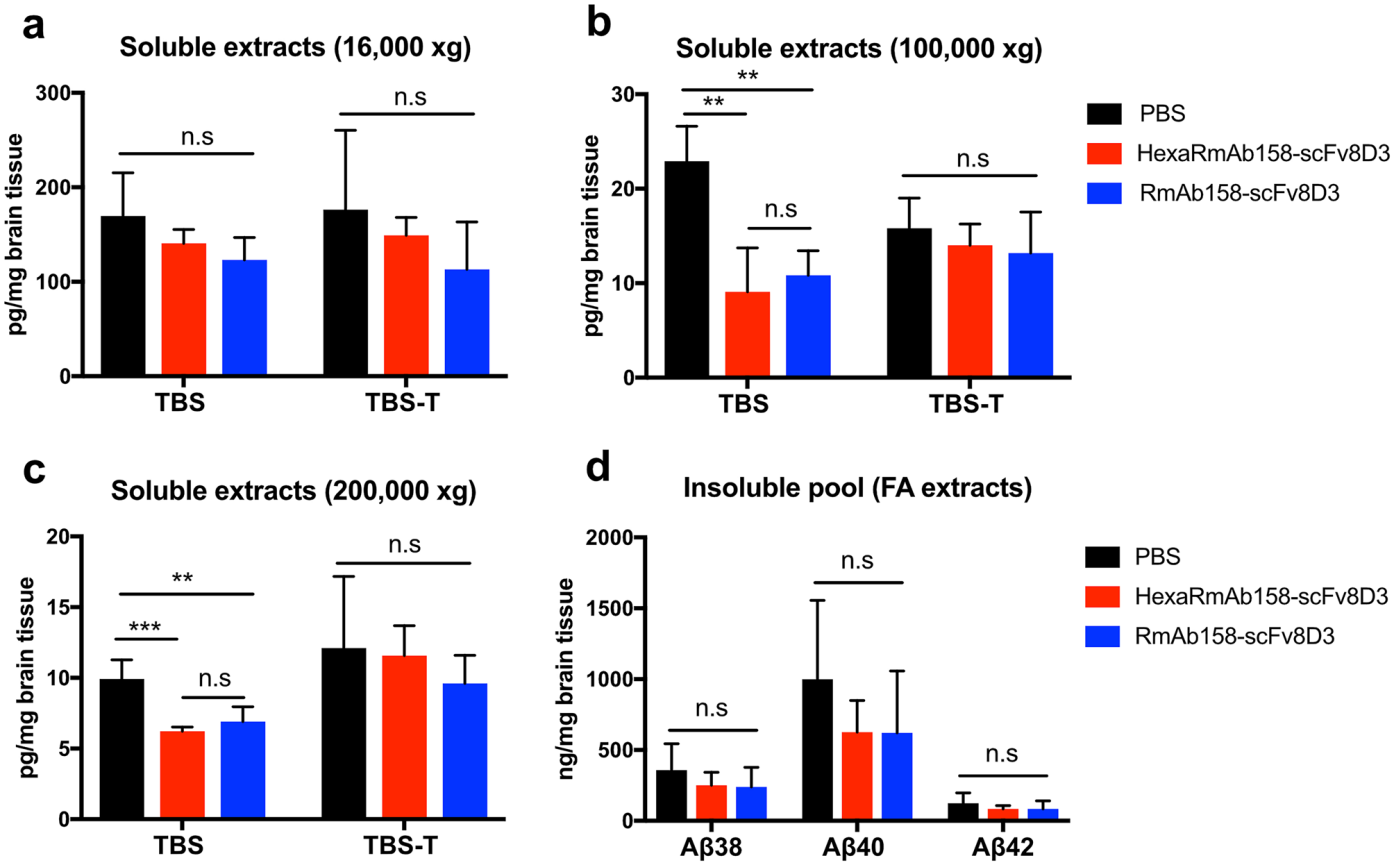
Concentration of soluble Aβ aggregates in the brain of tg-ArcSwe mice following treatment with therapeutic doses of 24 nmol/kg of HexaRmAb158-scFv8D3 or RmAb158-scFv8D3, using PBS as a negative control. **a** No significant differences were detected in concentration of soluble Aβ aggregates among the groups in TBS and TBS-T brain extracts centrifuged at 16,000 xg. **b** Concentration of soluble Aβ aggregates were significantly reduced in TBS soluble brain extracts ultra-centrifuged at 100,000 × g following treatment with HexaRmAb158-scFv8D3 and RmAb158-scFv8D3 compared to PBS. **c** Concentration of soluble Aβ aggregates were significantly reduced in TBS soluble brain extracts ultra-centrifuged at 200,000 × g following treatment with HexaRmAb158-scFv8D3 and RmAb158-scFv8D3 compared to PBS controls. **d** No significant differences were detected in total Aβ38, Aβ40 and Aβ42 concentration among the groups in FA brain extracts centrifuged at 16,000 xg. Results are presented as mean ± SD and analysed with one-way ANOVA followed by Bonferroni’s post-hoc analysis. (*: *p* < 0.05, **: *p* < 0.01, ***: *p* < 0.001, n.s.: *p* > 0.05), (*n* = 4/PBS, *n* = 5/HexaRmAb158-scFv8D3, *n* = 5/RmAb158-scFv8D3)

Aβ concentration was determined in post mortem brain tissue from tg-ArcSwe mice treated with therapeutic doses of HexaRmAb158-scFv8D3 or RmAb158-scFv8D3, using PBS as a negative control treatment. No significant differences were detected in soluble Aβ aggregate concentration, neither in TBS nor in TBS-T soluble fractions centrifuged at 16,000 × g between the three treatment groups (Fig. 7a). However, in the TBS soluble brain extracts ultra-centrifuged at 100,000 × g and 200,000 × g, Aβ aggregates were significantly decreased in mice treated with HexaRmAb158-scFv8D3 and RmAb158-scFv8D3 compared to PBS (Fig. 7b, c). No differences were detected in TBS-T extracts ultra-centrifuged at 100,000 × g and 200,000 × g among the groups (Fig. 7b, c). In addition, no significant differences were found in the concentration of total Aβ present in the FA soluble brain extracts after treatment (Fig. 7d). The biggest difference in treatment outcome between HexaRmAb158-scFv8D3 and RmAb158-scFv8D3 is expected to be in small aggregates [23]. Nevertheless, in tissue homogenates, it is very difficult to selectively quantify small aggregates since running them on, for instance, SDS or Native PAGE gels, can interfere with the aggregation state of Aβ and severely affect the results [45, 46].

A similar therapy study was also performed on 5.5 months old *App*^*NL−G−F*^ mice, with the exception that a lower antibody dose (12 nmol/kg) was used. None of the two antibody treatments displayed any significant effects on Aβ concentration (Supplementary Fig. 3). Finally, both antibody treatments displayed no effects on the concentration of pro-inflammatory cytokines in the brain and plasma of tg-ArcSwe mice compared to PBS (Supplementary Fig. 4).

## Discussion

Monoclonal antibodies targeting different species of Aβ have reached AD clinical trials. Some of these antibodies have been discontinued due to lack of efficacy, but three antibodies are currently investigated in phase 3 trials, and one was recently approved by the FDA [11, 19, 47]. Despite this recent success and reduction of brain Aβ, the beneficial effects of Aβ immunotherapy on cognition are still uncertain [13, 48]. Enhancing the BBB delivery and improving both antibody binding strength and selectivity to the toxic aggregates of Aβ could potentially enhance the therapeutic potential. We have previously introduced HexaRmA158 (Fig. 1c), a multivalent antibody with extra binding sites in the form of scFv, providing a 40 times enhanced avidity and slower rate of dissociation from Aβ protofibrils compared to RmAb158 [23]. Importantly, due to the short distance between the additional binding sites, the multivalent antibody could bind strongly and with avidity to small Aβ oligomers [23], an effect that has been difficult to achieve with conventional IgG antibodies [20–22]. HexaRmAb158 reduced neuronal cell death in vitro when added to cell cultures together with soluble Aβ aggregates [23], suggesting the potential of this antibody format to generate future therapeutic interventions targeting toxic Aβ aggregates in vivo.

The aim of the current study was to investigate the therapeutic potential of HexaRmAb158 with a TfR binding BBB transporter in vivo using preclinical mouse models of AD. The first part of the current study was to analyse the TfR binding and internalization of HexaRmAb158-scFv8D3 in vitro compared to that of RmAb158-scFv8D3. Using ELISA, we observed that the TfR binding of HexaRmAb158-scFv8D3 was twofold weaker compared to that of RmAb158-scFv8D3 (Fig. 2b). This could be attributed to the additional Aβ binding sites of HexaRmAb158-scFv8D3 that might sterically hinder the accessibility of scFv8D3 to TfR and cause the reduction in binding strength. Furthermore, both constructs were compared in an in vitro BBB transcytosis assay. Similar to the ELISA results, HexaRmAb158-scFv8D3 displayed a twofold decreased transport across the cEND endothelial cell layer compared to RmAb158-scFv8D3 (Fig. 3b), possibly due to the weaker TfR binding. These observations are also evident in vivo, as a higher concentration of HexaRmAb158-scFv8D3 was detected in the plasma compared to the blood pellet at the early time points (Fig. 5b, c). Stronger TfR binders are usually present at higher concentrations in the blood pellet [33, 49, 50], where red blood cells expressing TfR are present, while weaker binders (such as HexaRmAb158-scFv8D3 in our case) would be present at higher concentrations in the plasma.

Binding of HexaRmAb158 to in vivo generated soluble Aβ aggregates was evaluated in a sandwich ELISA setup for measuring the concentration of soluble Aβ aggregates in brain homogenates prepared from two AD mouse models (*App*^*NL−G−F*^ and tg-ArcSwe). Compared to RmAb158, the use of HexaRmAb158 as the capture antibody could detect higher concentrations of soluble Aβ aggregates in TBS and TBS-T soluble brain homogenates (Fig. 4a, b). The observed results can be related to the ability of HexaRmAb158 antibody to capture more Aβ aggregates due to the additional binding sites, but also due to the enhanced binding strength and slower dissociation [23]. However, when TBS soluble brain homogenates from *App*^*NL−G−F*^ mice were ultra-centrifuged at 100,000 × g, no differences were detected between the two antibodies, with the measured Aβ concentrations diminished substantially (Fig. 4c). This could be attributed to the size of Aβ aggregates in the supernatant after ultra-centrifugation at 100,000 × g, which might be smaller than the size range that these antibodies can ideally recognize [23, 36]. Interestingly, this was not the case for the ultra-centrifuged TBS soluble brain extracts from tg-ArcSwe mice, which could indicate that the size distribution of aggregates in the tg-ArcSwe model is different from that in the *App*^*NL−G−F*^ model. Here, HexaRmAb158 tended to detect higher Aβ concentrations compared to RmAb158 (Fig. 4c), probably due to its ability to detect and strongly bind small oligomers. The control antibody, 3D6, captured more soluble Aβ aggregates compared to HexaRmAb158 and RmAb158. 3D6 is the humanized version of bapineuzumab and binds equally well to all species of Aβ [51]. Hence, in TBS and TBS-T soluble brain extracts, 3D6 can detect not only oligomers and protofibrils (as it is the case for HexaRmAb158), but also other sizes of soluble Aβ aggregates starting from dimers and bigger. In these experiments, we were however not capable of characterizing which species of Aβ in tissue homogenates are being detected by these three antibodies. Commonly used methods such as SDS-PAGE and western blot have been shown to be associated with several drawbacks such as interference with oligomerization status of Aβ, enhancing Aβ fibrillization and smearing formation [45, 46].

The in vivo experiments were initiated to assess the ability of HexaRmAb158-scFv8D3 to cross the BBB and reach the brain in WT mice. High brain uptake (1.13% ID per gram tissue) was discovered following intravenous administration of HexaRmAb158-scFv8D3 at a tracer dose of 0.05 mg/kg. This was slightly lower than the brain uptake of RmAb158-scFv8D3 (1.47% ID per gram tissue) and in line with the in vitro BBB transcytosis result discussed in the previous section. Despite that, brain uptake of HexaRmAb158-scFv8D3 was around 60 times higher than what is observed for conventional antibodies without transporters [29, 52] and, thus, likely to be sufficient for the antibody to serve as an immunotherapeutic option in AD. Administration of the two antibodies at therapeutic doses of 12 nmol/kg led to lower brain uptake, suggesting a saturation of the TfR-mediated transcytosis mechanism as previously discussed [53], but still markedly higher than for conventional IgG antibodies.

One of the interesting observations in the current study was the detection of low concentrations of HexaRmAb158-scFv8D3 in the blood. Despite higher plasma concentration of HexaRmAb158-scFv8D3 at the early time points (Fig. 5b, c), a significantly lower blood and plasma concentration was found at the later time points, compared to RmAb158-scFv8D3 (Fig. 6c). In addition, blood samples collected at different time points throughout the study displayed a shorter blood half-life of HexaRmAb158-scFv8D3 (12.5 h), which is a twofold faster elimination than that of RmAb158-scFv8D3 (Fig. 6b). This could be considered a therapeutic limitation, as it will result in reduced systematic availability of the antibody. On the other hand, this can also be seen as an advantage, as lower antibody concentrations in the periphery could result in less off-target adverse effects. The low blood concentrations can also suggest the potential of this antibody format for diagnostic purposes as a brain-specific PET radioligand, as the concentrations in the brain are still high and similar to that of RmAb158-scFv8D3 (Fig. 6a). Several reasons for the concentration differences seen in blood may exist. For example, the multivalent antibody format has a weaker TfR binding capacity compared to RmAb158-scFv8D3 (Fig. 2b). TfR is present on red-blood cells [54] and binding of protein-drugs to TfR significantly enhances their blood half-life [34]. Another possible explanation can be attributed to the possibility of HexaRmAb158-scFv8D3 degradation in the periphery, due to the complexity of the design and the presence of the six additional scFv. The significantly higher concentrations of HexaRmAb158-scFv8D3 found in the kidneys at the early time points (Fig. 5d, e) and the lower concentrations in spleen at the later time points (Fig. 6d) could suggest antibody degradation.

Therapeutic potential of HexaRmAb158-scFv8D3 in tg-ArcSwe mice was demonstrated using a single intravenous injection of the antibody. A significant reduction in the most soluble Aβ aggregates (TBS soluble extracts ultra-centrifuged at 100,000 and 200,000 × g) was detected in mice treated with 24 nmol/kg HexaRmAb158-scFv8D3 (Fig. 7b, c). These ultra-centrifuged fractions contain the smallest soluble aggregates of Aβ, whose detection may be increased by the improved oligomer avidity of HexaRmAb158 [23]. The same effects were seen following treatment with RmAb158-scFv8D3 (Fig. 7b, c), similar to what has been displayed previously [44]. In the brain of 10-month-old tg-ArcSwe mice, the TBS-soluble fraction constitutes only around 0.01% of the total Aβ [55]. Despite that, this fraction contains the most toxic Aβ species (soluble oligomers and protofibrils) that are thought to cause dysfunction and loss of synapses which is one possible reason to the loss of cognition. It has been shown that there is a poor correlation between the amount of insoluble amyloid beta aggregates and AD progression [56, 57]. Hence, targeting the soluble toxic Aβ aggregates with our hexavalent antibody (having an improved binding selectivity towards these aggregates) can be of great therapeutic potential throughout the disease progression. No Aβ-lowering was detected in the soluble brain extracts centrifuged at 16,000 × g (Fig. 7a). This fraction still contains soluble Aβ aggregates, but also bigger and less diffusible aggregates [58] that are likely difficult to clear with a short-term, single dose study. As expected, antibody treatment displayed no Aβ lowering effects in the FA soluble brain extracts (Fig. 7d). This fraction contains total Aβ including plaque-deposited Aβ, to which both antibodies have only a moderate binding affinity [23, 36, 59]. Furthermore, both antibody treatments demonstrated no effects on the concentration of pro-inflammatory cytokines (Supplementary Fig. 4). A longer treatment study might be needed to assess the effects on inflammation, which is considered an integral part of AD pathophysiology.

In the current study, both antibody formats were essentially equal in lowering the concentration of soluble Aβ aggregates in vivo. The multivalent antibody format (HexaRmAb158) has previously demonstrated a stronger binding than RmAb158 to Aβ oligomers in vitro [23]. These species of Aβ should be highly present in the TBS soluble brain extracts ultra-centrifuged at 100,000 × g and 200,000 × g. However, these fractions might also contain soluble Aβ aggregates other than oligomers, such as the bigger protofibrils [60] or even smaller aggregates like trimers/dimers [61]. Therefore, other methods that could separate oligomers from other Aβ species in biological samples need to be developed. Analysing the size of Aβ aggregates on SDS PAGE or Native PAGE is not a suitable method since the such methods substantially alter the size of the aggregates. Further, longer treatment studies with multiple injections of the antibodies might be necessary to be able to detect differences in Aβ concentration in the different brain extracts. This study was designed as a proof-of-concept study for demonstrating the therapeutic potential of HexaRmAb158-scFv8D3. Despite the less efficient BBB delivery of the multivalent antibody format, brain retention at 72 h post injection and clearance of soluble Aβ aggregates were equal to those obtained with RmAb158-scFv8D3 treatment, suggesting an even more efficient in vivo target engagement of HexaRmAb158-scFv8D3. Our previous in vitro study demonstrated a stronger binding of HexaRmAb158 to Aβ oligomers compared to RmAb158 [23]. However, it is difficult to selectively isolate and measure Aβ oligomers in brain homogenates in vivo. Therefore, despite the similar effects in clearing soluble Aβ aggregates between the two antibodies in vivo, we think that HexaRmAb158-scFv8D3 has a higher potential than RmAb158-scFv8D3 in future development of therapies for AD.

Two AD mouse models were used in this study, tg-ArcSwe and *App*^*NL−G−F*^. However, the reduction of Aβ following antibody treatment was only detected in the tg-ArcSwe model, in line with what has been demonstrated previously [44]. This can be related to different Aβ aggregation processes in the two mouse models, which is faster in the *App*^*NL−G−F*^ mice [62–64]. A faster aggregation process could possibly lead to less small aggregates being present compared to larger aggregates. Therefore, the nature and size of Aβ aggregates that are present in the different brain extracts centrifuged at different speeds may be different between the two models. In addition, the negative outcomes seen in *App*^*NL−G−F*^ mice can be related to dosing, as a 50% reduced treatment dose was administered to these mice compared to the therapy study performed on tg-ArcSwe mice.

## Conclusion

By recombinantly linking the hexavalent antibody (HexaRmAb158) and the BBB transporter moiety (scFv8D3), this study demonstrates successful delivery of HexaRmAb158-scFv8D3 through the BBB into the brain. The ability of the bispecific multivalent antibody format to decrease the levels of soluble Aβ aggregates in tg-ArcSwe mice highlight its therapeutic potential in AD. The design can be applied to generate other antibody treatments capable of passing the BBB and binding to other pathological protein aggregates such as α-synuclein in Parkinson’s disease.

## Supplementary Information

Below is the link to the electronic supplementary material.

Supplementary file1 (DOCX 819 kb)

Supplementary file2 (PDF 3045 kb)

Supplementary file3 (PDF 3063 kb)

Supplementary file4 (PDF 1727 kb)

Supplementary file5 (PDF 713 kb)

Supplementary file6 (PDF 1984 kb)

Supplementary file7 (PDF 284 kb)

Supplementary file8 (PDF 285 kb)

Supplementary file9 (PDF 284 kb)

## Data Availability

The datasets used and/or analysed during the current study are available from the corresponding author on reasonable request.

## Abbreviations

Aβ: Amyloid-beta
AD: Alzheimer’s disease
APP: Amyloid precursor protein
Arc: Arctic APP mutation
BBB: Blood–brain barrier
scFv: Single-chain fragment variable
Swe: Swedish APP mutation
TfR: Transferrin receptor
WT: Wild-type
