# Supplementary material for: A Brain-Targeting Bispecific-Multivalent Antibody Clears Soluble Amyloid-Beta Aggregates in Alzheimer’s Disease Mice
Source: Neurotherapeutics. 2022 Aug 8;19(5):1588–602. doi: 10.1007/s13311-022-01283-y (PMC9606191; doi:10.1007/s13311-022-01283-y)
Supplement: Supplementary file 1 — Supplementary file1 (DOCX 819 kb) [file 13311_2022_1283_MOESM1_ESM.docx]

**Supplementary information**

**
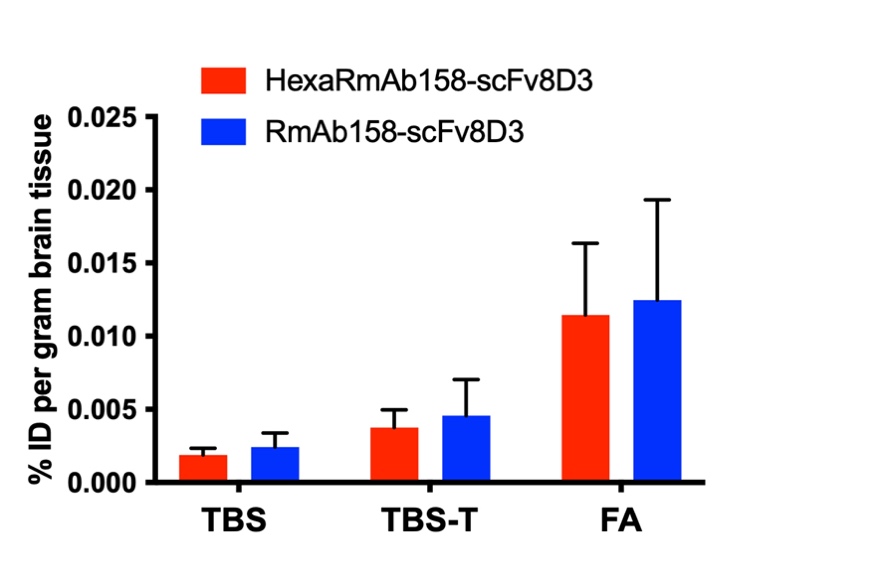
**

**Supplementary Fig. 1** Comparison of [^125^I]HexaRmAb158-scFv8D3 and [^125^I]RmAb158-scFv8D3 concentrations in three brain extracts of Aβ in tg-ArcSwe mice at 72 h post injection of equimolar therapeutic doses of 24 nmol/kg of the antibodies. Both antibodies mainly retained in the FA-soluble brain extracts (containing insoluble fibrils and plaques) compared to TBS (soluble) and TBS-T (membrane bound) extracts of Aβ. Results are presented as mean ± SD, (n=5 per group).


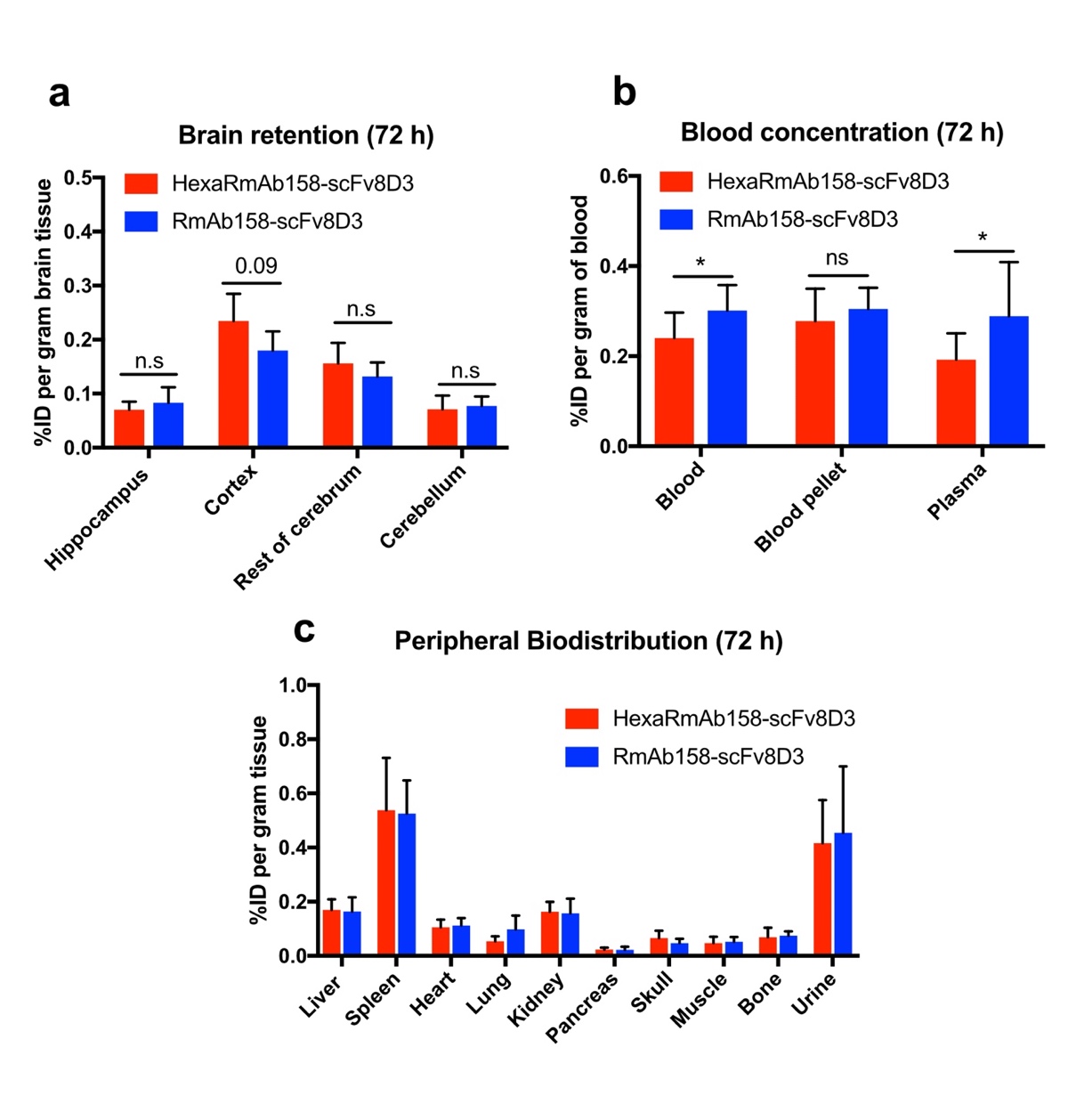


**Supplementary Fig. 2** Comparison of [^125^I]HexaRmAb158-scFv8D3 and [^125^I]RmAb158-scFv8D3 concentrations in the brain, blood and peripheral organs of 5.5-months old App^NL-G-F^ mice at 72 h post injection of equimolar therapeutic doses of 2.5 mg/kg of the antibodies. **(a)** Retention of the two antibodies in brain regions, expressed as percentage of injected dose (% ID) per gram brain tissue, measured 72 h post injection. No statistically significant differences detected between the two groups. A trend to a higher retention (p value 0.09) detected in the cortex of mice treated with [^125^I]HexaRmAb158-scFv8D3 compared to [^125^I]RmAb158-scFv8D3 treated mice. **(b)** Statistically significant decrease in the plasma concentrations of [^125^I]HexaRmAb158-scFv8D3 compared to [^125^I]RmAb158-scFv8D3, 72 h post injection. **(c)** Similar retention of [^125^I]HexaRmAb158-scFv8D3 and [^125^I]RmAb158-scFv8D3 in the peripheral organs, 72 h post injection. Results are presented as mean + SD and analysed with unpaired t-test. (*: p < 0.05, **: p < 0.01, ***: p < 0.001, n.s: p > 0.05), (n=9/HexaRmAb158-scFv8D3; n=8/RmAb158-scFv8D3)


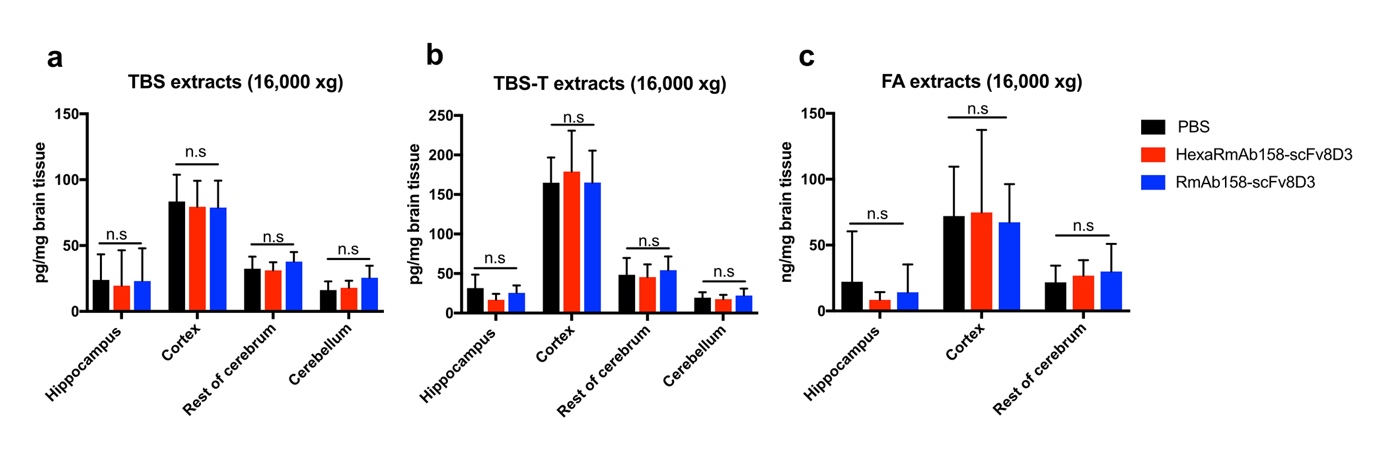


**Supplementary Fig. 3** Concentration of Aβ in the brain regions of App^NL-G-F^ mice following treatment with therapeutic doses of 12 nmol/kg of HexaRmAb158-scFv8D3 or RmAb158-scFv8D3, using PBS as a negative control. No significant differences detected in Aβ concentration among the groups in **(a)** TBS soluble, **(b)** TBS-T soluble or **(c)** FA soluble brain extracts centrifuged at 16,000 xg. A trend towards a decrease (p value 0.06) in Aβ concentration was detected in the TBS-T hippocampus extractions of HexaRmAb158-scFv8D3 treated mice. Results are presented as mean + SD and analysed with one-way ANOVA followed by Bonferroni’s post-hoc analysis. (*: p < 0.05, **: p < 0.01, ***: p < 0.001, n.s: p > 0.05), (n=8/PBS, n=9/HexaRmAb158-scFv8D3, n=8/RmAb158-scFv8D3)


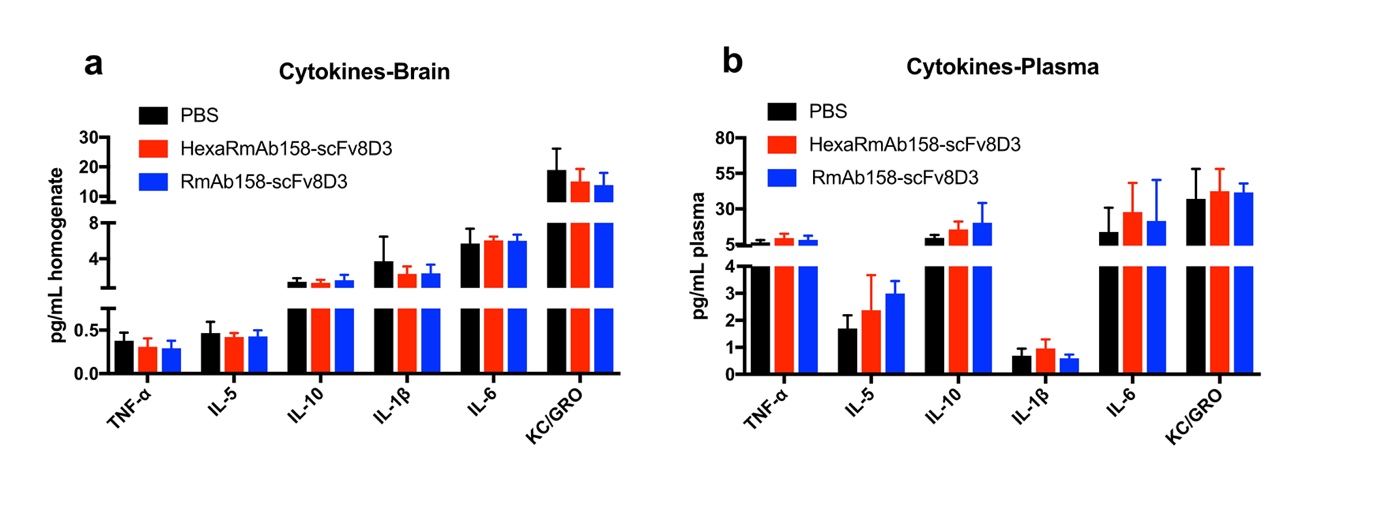


**Supplementary Fig. 4** Concentration of cytokines in the brain and plasma of tg-ArcSwe mice following treatment with therapeutic doses of 24 nmol/kg of HexaRmAb158-scFv8D3 or RmAb158-scFv8D3, using PBS as a negative control. No significant differences detected in cytokines concentration among the groups in **(a)** TBS soluble brain extracts and **(b)** plasma. V-PLEX proinflammatory panel (K15048D) was used for quantification. Results are presented as mean + SD and analysed with one-way ANOVA followed by Bonferroni’s post-hoc analysis. (*: p < 0.05, **: p < 0.01, ***: p < 0.001, n.s: p > 0.05), (n=4/PBS, n=5/HexaRmAb158-scFv8D3, n=5/RmAb158-scFv8D3)

**Supplementary Fig. 5** Full picture of the SDS-PAGE gel presented in Fig. 2a
